# Incidence and frequency of cancer cachexia during chemotherapy for advanced pancreatic ductal adenocarcinoma

**DOI:** 10.1007/s00520-020-05346-8

**Published:** 2020-02-26

**Authors:** Shuichi Mitsunaga, Eiji Kasamatsu, Koji Machii

**Affiliations:** 1 Department of Hepatobiliary and Pancreatic Oncology, National Cancer Center, National Cancer Center Hospital East, 6-5-1, Kashiwanoha, Kashiwa, Chiba, 277-8577 Japan; 2 Division of Biomarker Discovery, Exploratory Oncology Research & Clinical Trial Center, National Cancer Center, Chiba, Japan; 3 Medical Affairs Department, Ono Pharmaceutical Co., Ltd, Osaka, Japan

**Keywords:** Cachexia, Incidence, Survival, Pancreatic cancer, Adverse events

## Abstract

**Purpose:**

Cachexia influences the patient’s physical wellbeing and quality of life, and the patient’s ability to tolerate their cancer therapies, especially cytotoxic chemotherapy. The purpose of this study was to investigate the frequency and timing of onset of cancer cachexia during chemotherapy and its association with prognosis and toxicity in patients with pancreatic ductal adenocarcinoma (PDAC).

**Methods:**

We performed a retrospective study in patients who underwent first-line chemotherapy after diagnosis of advanced PDAC between 6 June 2008 and 31 March 2017. Base cachexia (weight loss up to 6 months before starting first-line chemotherapy) and follow-up cachexia (after starting first-line chemotherapy) were defined as weight loss > 2% with a body mass index (BMI) < 20 kg/m^2^ or weight loss > 5%.

**Results:**

A total of 150 patients were registered. The median age and BMI were 65 years and 21.7 kg/m^2^, respectively. Base cachexia occurred in 50% of patients. Follow-up cachexia occurred in 32% within 12 weeks of starting first-line chemotherapy, reaching 64% at 1 year. Overall survival was not significantly different between patients with and without follow-up cachexia, regardless of whether cancer cachexia occurred within 12, 24, or 48 weeks of starting first-line treatment. Appetite loss, fatigue, nausea, and diarrhea were more frequent in patients with follow-up cachexia than in those without follow-up cachexia.

**Conclusion:**

Follow-up cachexia had an early onset, but was not a prognostic factor for overall survival in patients with PDAC. Some adverse events tended to be more frequent in patients with follow-up cachexia than in those without follow-up cachexia.

**Electronic supplementary material:**

The online version of this article (10.1007/s00520-020-05346-8) contains supplementary material, which is available to authorized users.

## Introduction

Cancer cachexia is a multi-factorial metabolic syndrome characterized by ongoing loss of skeletal muscle mass that cannot be fully reversed by conventional nutritional support and leads to progressive functional impairment. Its pathophysiology is characterized by a hypercatabolic state driven by reduced food intake and abnormal metabolism [1]. It is now becoming clear that cancer cachexia has an impact on the patient’s physical wellbeing and quality of life, and the patient’s ability to tolerate their cancer therapies, especially cytotoxic chemotherapy [2–6]. The definitions of cancer cachexia and the diagnostic criteria differed between trials in the past [7, 8]. In 2011, the European Palliative Care Research Collaborative (EPCRC) proposed the definition of cancer cachexia, as (a) weight loss > 5% over the past 6 months, (b) weight loss of > 2% and body mass index (BMI) < 20 kg/m^2^, or (c) weight loss of > 2% and diagnosis of sarcopenia [1]. The EPCRC definition has now been accepted as the current consensus of cancer cachexia.

In a study of untreated non-small cell lung cancer (NSCLC) patients with a 52-week observation period, weight loss of  ≥ 5% was observed in ≥ 20% of registered patients, and weight loss was associated with decreases in Karnofsky performance scale and quality of life, and with shortened survival [9]. In a study of pancreatic ductal adenocarcinoma (PDAC), the incidence of base cachexia, defined as weight loss > 5% over up to 6 months prior to diagnosis, was 63% and base cachexia was found to be a prognostic factor for reduced overall survival (OS) [10]. In a study of several types of cancer that assessed cachexia during chemotherapy of 191 patients, unintentional weight loss was reported in over 63% of patients, with weight loss of ≥ 5% and ≥ 10% in 38.7% and 24.6%, respectively, and weight loss was associated with gastrointestinal symptoms [11]. Despite these findings, very few reports have described the frequency or prognosis of cachexia during cancer treatment, and none have focused on PDAC. Therefore, we performed this retrospective study in order to evaluate the frequency and timing of onset of EPCRC-based cachexia during chemotherapy and its association with prognosis or associated toxicities. These data will be valuable for the management of patients with advanced PDAC.

## Methods

### Ethics

This study was approved by the ethics review committee of the National Cancer Center Hospital East (reference 2018-148). This study was registered on the University Hospital Medical Information Network-Clinical Trials Registry (UMIN000034972).

### Patients

The medical records database was searched to retrieve the records of patients who were clinically and pathologically diagnosed with advanced PDAC and who underwent first-line systemic chemotherapy between 6 June 2008 and 31 March 2017 at the National Cancer Center Hospital East.

### Definition of cancer cachexia

In this study, we defined cancer cachexia as either weight loss > 5% or weight loss > 2% with a BMI < 20 kg/m^2^. Base cachexia was defined as weight loss within 6 months before the start of chemotherapy. Follow-up cachexia was defined as cachexia that occurred after the start of first-line systemic chemotherapy, based on the change in body weight from the start of chemotherapy at the following observation times: 1–12, 13–24, 25–36, 37–48, and beyond 48 weeks.

### Data collection

Data on body weight, laboratory tests, and toxicities (Common Terminology Criteria for Adverse Events; CTCAE version 4.0) [12] were collected at the start of chemotherapy (0 weeks). The latest body weight, laboratory test data, and worst AE data were collected in 4-week periods in the observation time. The greatest body weight up to 6 months before the start of chemotherapy was also collected.

### Data analyses

The primary endpoints were the timing of cachexia onset (1–12, 13–24, 25–36, 37–48, and beyond 48 weeks) and the cumulative incidence of follow-up cachexia from the start of first-line chemotherapy to 156 weeks. The cumulative incidence of follow-up cachexia was calculated as the number of new episodes of follow-up cachexia without considering death as a competing risk.

Secondary endpoints were the associations between follow-up cachexia and the frequency of adverse events (AEs), OS, treatment status, and laboratory variables. AEs were categorized using CTCAE version 4.0 of the National Cancer Institute [12].

OS was calculated from the beginning of first-line chemotherapy. Survival curves were drawn using the Kaplan–Meier method. OS was compared using the log-rank test between patients who developed follow-up cachexia within 12, 24, and 48 weeks of starting first-line chemotherapy and patients who did not develop follow-up cachexia. Survival curves were also drawn by landmark analyses at 12, 24, and 48 weeks.

We originally planned to analyze data according to the presence or absence of follow-up cachexia within 48 weeks of starting first-line treatment. Owing to the disparity in the number of patients, we also performed exploratory analyses in which patients were divided according to whether they experienced follow-up cachexia within 12 weeks or 24 weeks of starting first-line chemotherapy.

Hazard ratios (HR) with 95% confidence intervals (CI) according to the presence or absence of follow-up cancer cachexia for OS were evaluated using the Cox proportional hazard model with or without adjustment for age, sex, multidrug therapy, Eastern Cooperative Oncology Group (ECOG) performance status, Union for International Cancer Control (UICC) stage, C-reactive protein (CRP), base cachexia, and carbohydrate antigen 19-9 (CA19-9). The cut-off period for the occurrence of follow-up cachexia was set at 24 weeks because the numbers of patients with and without cachexia by this time-point were similar.

Median survival times were calculated with 95% confidence intervals (CI), which were determined using the Brookmeyer and Crowley method.

All *P* values were two-sided, and *P* < 0.05 was considered statistically significant. Data analyses were performed using SAS for Windows version 9.4 or later (SAS Institute, Cary, NC, USA).

## Results

### Patients

A total of 150 patients were identified and included in this study, of which 88 patients (58.7%) were male and 62 patients (41.3%) were female (Table 1). The median (range) age and BMI were 65 (35–83) years and 21.7 (13.8–33.3) kg/m^2^, respectively. The Eastern Cooperative Oncology Group performance status (ECOG PS) scores were 0 in 70.7% and 1 in 29.3%. The primary tumor sites were the pancreatic head (40.0%) and body (42.0%), and most patients had stage IV cancer (70.7%). First-line chemotherapy was modified FOLFIRINOX (30.0%), gemcitabine monotherapy (38.0%), or gemcitabine + nab-paclitaxel (32.0%). The median (range) albumin, CRP, and hemoglobin were 4.0 (2.7–4.9) g/dL, 0.39 (0.01–9.94) mg/dL, and 12.6 (8.1–18.2) g/dL, respectively. Base cachexia was found in 75 patients (50.0%).

**Table 1:** Patient characteristics

	Value
*N*	150
Sex	
Male	88 (58.7%)
Female	62 (41.3%)
Age, years	65 (35–83)
BMI, kg/m^2^	21.7 (13.8–33.3)
ECOG PS	0 (0–1)
0	106 (70.7%)
1	44 (29.3%)
Primary site	
Pancreatic head	60 (40.0%)
Pancreatic body	63 (42.0%)
Pancreatic tail	27 (18.0%)
UICC stage	
III	44 (29.3%)
IV	106 (70.7%)
Modified Glasgow prognosis score^a^	
A	96 (64.0%)
B	7 (4.7%)
C	38 (25.3%)
D	9 (6.0%)
First-line chemotherapy	
Modified FOLFIRINOX	45 (30.0%)
Gemcitabine monotherapy	57 (38.0%)
Gemcitabine + nab-paclitaxel	48 (32.0%)
Cancer cachexia at the start of first-line chemotherapy (base cachexia), yes	75 (50.0%)
Comorbidities	
Hypertension	46 (30.7%)
Diabetes mellitus	38 (25.3%)
Dyslipidemia	9 (6.0%)
Other	22 (14.7%)
None	73 (48.7%)
CrCl^b^, mL/min	84.26 (36.30–177.05)
Neutrophil/lymphocyte ratio	2.95 (0.81–12.68)
CA19-9, U/mL	747.9 (0.2–284,200)
Sodium, mmol/L	141 (131–144)
Potassium, mmol/L	4.3 (3.4–6.3)
AST, U/L	22 (11–136)
ALT, U/L	23 (7–187)
ALP, U/L	321 (104–2558)
Cholinesterase, U/L	268 (109–553)
Total bilirubin, mg/dL	0.65 (0.12–2.20)
Neutrophil count, cells/μL	3805 (1510–12,650)
WBC count, cells/μL	5900 (2500–16,100)
Lymphocyte count, cells/μL	1310 (440–2770)
Platelet count, × 10^4^ cells/μL	18.6 (8.5–52.4)
Creatinine, mg/dL	0.66 (0.33–1.34)
Albumin, g/dL	4.0 (2.7–4.9)
Total protein, g/dL	6.9 (5.6–8.1)
CRP, mg/dL	0.39 (0.01–9.94)
Hemoglobin, g/dL	12.6 (8.1–18.2)
Glucose, mg/dL	109 (59–350)

Values are number (percent) of patients or median (range)

*BMI* body mass index, *ECOG PS* Eastern Cooperative Oncology Group performance status, *UICC* Union for International Cancer Control, *CrCl* creatinine clearance, *CA19*-*9* carbohydrate antigen 19-9, *AST* aspartate aminotransferase, *ALT* alanine aminotransferase, *ALP* alkaline phosphatase, *WBC* white blood cell count, *CRP* C-reactive protein

^a^A = albumin ≥ 3.5 g/dL and CRP < 1.0 mg/dL; B = albumin < 3.5 g/dL and CRP < 1.0 mg/dL; C = albumin ≥ 3.5 g/dL and CRP ≥ 1.0 mg/dL; D = albumin < 3.5 g/dL and CRP ≥ 1.0 mg/dL

^b^Cockcroft–Gault formula

### Frequency and timing of follow-up cachexia

**Fig. 1:**
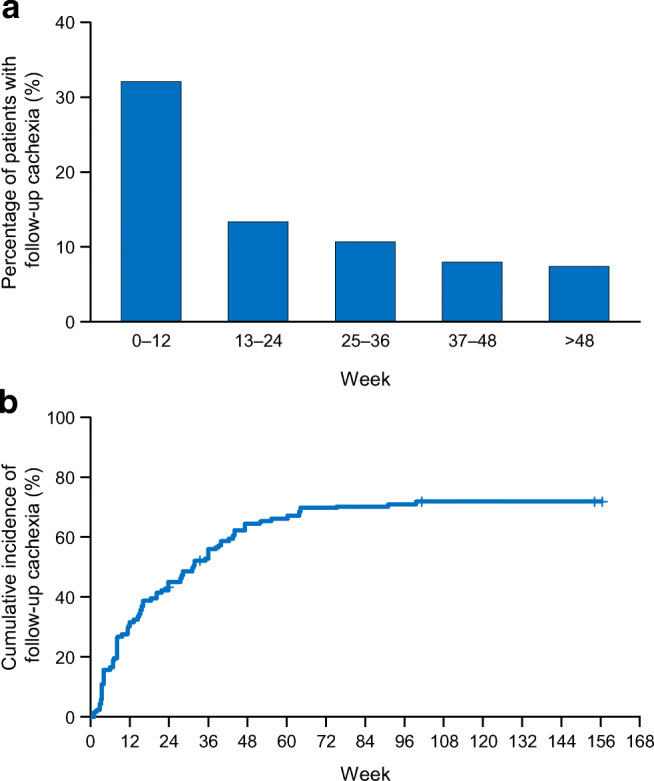
Timing of onset (**a**) and cumulative incidence (**b**) of follow-up cachexia after the start of first-line chemotherapy

Figure 1a shows the timing of follow-up cachexia onset. A total of 32.0% of patients experienced cachexia within 12 weeks of starting chemotherapy, while 13.3% experienced cachexia at 13–24 weeks, 10.7% at 25–36 weeks, and 8.0% at 37–48 weeks. Figure 1b shows the cumulative incidence of follow-up cachexia after the start of first-line chemotherapy. The cumulative incidence was 45.3% at 24 weeks, 64.0% at 48 weeks, and 71.3% over the full study period. Follow-up cachexia occurred during first-line chemotherapy in 65 patients (43.3%) (their characteristics are summarized in Table S1). Of 75 patients with base cachexia, 38 experienced follow-up cachexia during first-line chemotherapy (50.7%), while the other 37 did not. Of 75 patients without base cachexia, 27 experienced follow-up cachexia during first-line therapy (36.0%) (Table S1).

### Survival

**Fig. 2:**
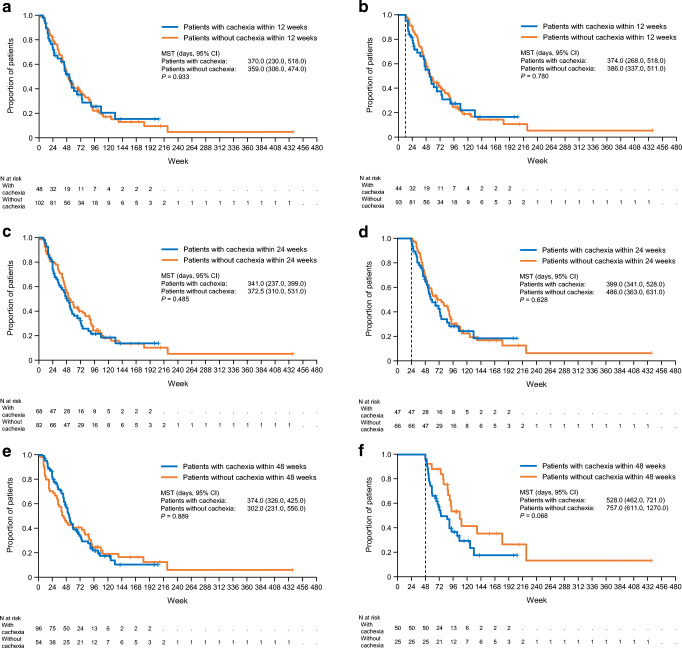
Overall survival according to presence or absence of follow-up cachexia within 12 (**a**), 24 (**c**), or 48 (**e**) weeks and at the landmark analyses at 12 (**b**), 24 (**d**), and 48 (**f**) weeks after the start of chemotherapy. *CI* confidence interval, *MST* median survival time

Figure 2 shows the OS for patients according to whether they experienced follow-up cachexia (or not) within 12, 24, or 48 weeks of starting chemotherapy. Within 12 weeks from the start of first-line chemotherapy, there was no difference in OS between patients with and without cachexia (Fig. 2a). The median survival time (95% CI) was 370 (230–518) days for patients with cachexia versus 359 (306–474) days for patients without cachexia. There were no differences in OS between patients with or without cachexia when we divided the patients according to whether they experienced follow-up cachexia within 24 weeks (Fig. 2c) or 48 weeks (Fig. 2e) of starting chemotherapy. Landmark analyses were also done at these time-points. In the landmark analysis at 48 weeks, the median survival time in patients without follow-up cachexia (757 vs. 528 days) was slightly longer than that in patients with follow-up cachexia, although this was not statistically significant (log-rank *P* = 0.068) (Fig. 2f). In the landmark analyses at 12 and 24 weeks, median OS was not significantly different between patients with or without cachexia (Fig. 2b, d). Follow-up cachexia occurring within 24 weeks of starting first-line chemotherapy was not a prognostic factor for OS in the unadjusted model (HR 1.12, 95% CI 0.72–1.74, *P* = 0.628) or in the adjusted model (Table 2). CRP and multidrug therapy were prognostic factors for OS in the adjusted model. Base cachexia was not a prognostic factor for OS in the adjusted model.

**Table 2:** Prognostic factors for overall survival by Cox proportional hazard model analysis

	HR (95% CI)	*P* value
Presence of follow-up cachexia within 24 weeks of starting first-line chemotherapy (yes vs. no)	1.12 (0.70–1.79)	0.637
Age	1.00 (0.98–1.02)	0.945
Sex (female vs. male)	1.18 (0.75–1.85)	0.480
Multidrug therapy (vs. monotherapy)	0.29 (0.17–0.48)	< 0.001
ECOG PS (1 vs. 0)	1.39 (0.81–2.39)	0.228
UICC stage (IV vs. III)	1.43 (0.87–2.35)	0.157
CRP	1.14 (1.01–1.30)	0.039
Body weight loss at start of first-line chemotherapy (base cachexia; yes vs. no)	1.18 (0.74–1.87)	0.495
CA19-9 (positive vs. negative)	0.97 (0.58–1.63)	0.920

*HR* hazard ratio, *CI* confidence interval, *ECOG PS* Eastern Cooperative Oncology Group performance status, *UICC* Union for International Cancer Control, *CRP* C-reactive protein, *CA19*-9 carbohydrate antigen 19-9

### AEs in patients with or without follow-up cachexia

**Fig. 3:**
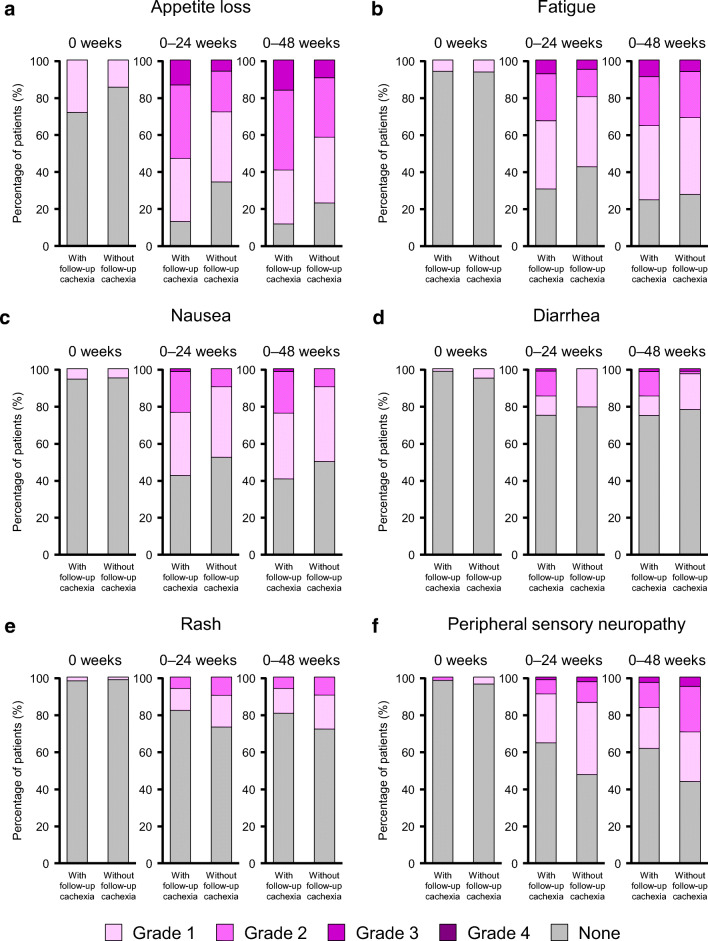

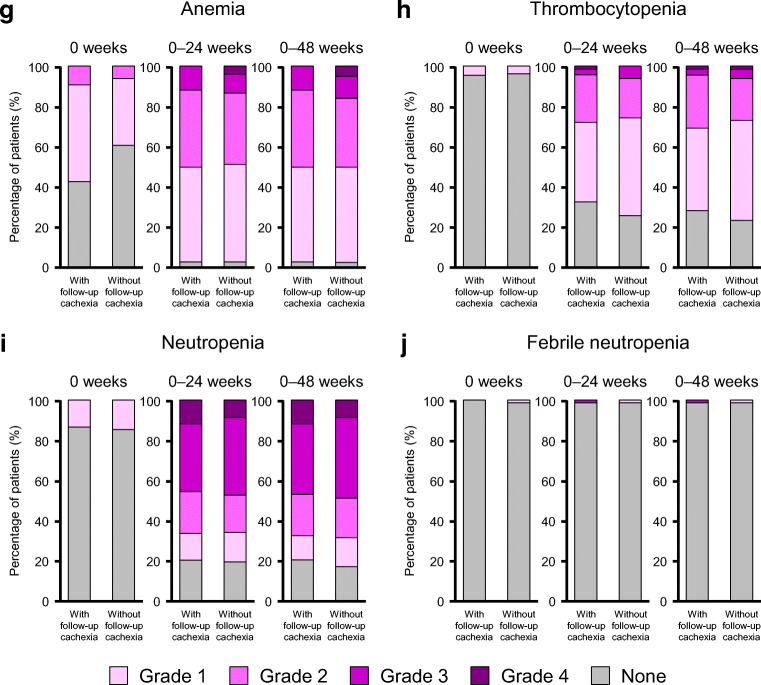
Frequency and grade of adverse events in patients with or without follow-up cachexia up to 0, 24, or 48 weeks after starting first-line chemotherapy for the following adverse events: appetite loss (**a**), fatigue (**b**), nausea (**c**), diarrhea (**d**), rash (**e**), peripheral sensory neuropathy (**f**), anemia (**g**), thrombocytopenia (**h**), neutropenia (**i**), and febrile neutropenia (**j**)

Figure 3 shows the frequency, timing, and grade of AEs in patients with or without follow-up cachexia (cachexia occurring within 24 weeks after the start of first-line chemotherapy).

The rates of AEs were generally low at baseline (week 0) except for appetite loss, which was found in ca. 28% of patients with follow-up cachexia and 15% of patients without follow-up cachexia (Fig. 3a). As indicated in these figures, there were marked increases in the rate of grade ≥ 2 AEs, especially appetite loss (Fig. 3a), fatigue (Fig. 3b), nausea (Fig. 3c), and diarrhea (Fig. 3d), in patients with follow-up cachexia. The frequency and grade of rash and peripheral sensory neuropathy also tended to increase over time (Fig. 3e, f).

Anemia was reported in 57% of patients with follow-up cachexia (9% with grade 2 and 49% with grade 1), and in 39% of patients without follow-up cachexia (6% with grade 2 and 33% with grade 1) (Fig. 3g). Thrombocytopenia and neutropenia AEs were rare at week 0 (Fig. 3h, i). There were marked increases in the frequencies and grades of anemia, thrombocytopenia, and neutropenia in patients with or without follow-up cachexia (Fig. 3g, h, i). The incidence of febrile neutropenia remained low throughout the follow-up period (Fig. 3j).

### Laboratory variables

There was a small increase in CRP levels at 48 weeks in patients with follow-up cachexia (median change: + 0.280 mg/dL) and in patients without (median change + 0.580 mg/dL), which was significantly different (*P* = 0.044) (Table S2). There was a small decrease in albumin over the same period of time in both groups, with a median change of − 0.30 g/dL in patients with follow-up cachexia and − 0.55 g/dL in patients without follow-up cachexia. There were decreases in neutrophil and total lymphocyte counts in patients with follow-up cachexia, while the neutrophil count increased and total lymphocyte count decreased in patients without follow-up cachexia. Other than the change in CRP levels at 48 weeks, there were no significant differences in changes of these laboratory variables between the two groups.

## Discussion

Key findings of this study include the high rates of follow-up cachexia in patients with advanced pancreatic cancer, and that about one-third of patients experienced follow-up cachexia within 12 weeks of starting first-line chemotherapy, increasing up to 45% by 24 weeks. These findings are similar to those in a prior report of patients with lung cancer [13]. The cumulative incidence of follow-up cachexia was 71% over the study period. However, because deaths as a competing risk were not used to censor the analysis, the results suggest that all patients might experience follow-up cachexia.

The median survival time was not significantly different between patients with and without follow-up cachexia, regardless of whether cachexia occurred within 12, 24, or 48 weeks of starting first-line chemotherapy. This result was similar to the previous report in pancreatic cancer, although the definition of cachexia was different from this study [14]. In our study, we found that multidrug therapy (vs. monotherapy) was a significant prognostic factor. A recent study indicated that the effect of base cachexia on survival could be modulated by chemotherapy in patients with pancreatic cancer, although base cachexia was a significant prognostic factor [10]. These findings suggest that the survival benefit provided by chemotherapy may reduce the negative impact of follow-up cachexia on survival.

As expected, the frequency and grade of AEs tended to increase over time after starting first-line chemotherapy (Fig. 3). AEs were frequent in both patients with and without follow-up cachexia. Notably, the rates of some AEs with grade ≥ 2, particularly appetite loss, fatigue, nausea, and diarrhea, were high in patients with follow-up cachexia, while the frequencies and grades of anemia, thrombocytopenia, and neutropenia showed similar increases in patients with and without cachexia. Although the cause–effect relationship between cachexia and the onset of appetite loss and fatigue is not fully clear, the higher frequency and grade of appetite loss and fatigue may interfere with the patient’s ability to continue chemotherapy or affect the patient’s wellbeing and quality of life. In a clinical trial in which patients were treated with gemcitabine + nab-paclitaxel for pancreatic cancer, it was reported that the treatment regimen was discontinued in 10% of patients due to an AE and in 20% of patients due to unacceptable toxicity [15]. In patients with gastrointestinal malignancies, it was reported that those experiencing weight loss generally had worse outcomes, partly because they received less chemotherapy and developed more toxicity [14]. Therefore, it is important to carefully monitor the body weight of patients during chemotherapy.

We also noted that follow-up cachexia was relatively common in patients with base cachexia. This may reflect the high tendency for continuation of past weight loss after starting chemotherapy. Therefore, physicians should also pay attention to the changes in body weight that occurred before the start of chemotherapy.

Finally, in the present study, we adopted two EPCRC definitions because information on sarcopenia was unavailable. The cachexia criteria used here may not be generalizable to all patient populations or ethnic groups owing to differences in build, lifestyle, dietary habits, and background metabolic rate, for example [16]. Alternatively, it may be possible to use Evans’ diagnostic criteria, which take account of inflammatory markers and poor appetite [17].

### Limitations

A limitation of this study is that multiple first-line chemotherapy regimens were used, which affect OS and the rates/types of AEs. Thus, these regimens may have different associations with frequency or impact of cachexia. Other limitations include its retrospective single-center design and that base cachexia may influence the outcomes reported here. In addition, although we found a high rate of AEs during chemotherapy, the design of our study means we could not determine whether follow-up cachexia contributed to the onset and grade of the AEs, or vice versa.

## Conclusions

In conclusion, the incidence of follow-up cachexia was highest within 12 weeks of starting first-line chemotherapy in this cohort of patients with advanced PDAC. Follow-up cancer cachexia occurred in 64% of patients within 48 weeks of starting first-line chemotherapy. However, it was not a prognostic factor for OS. The frequency and grade of appetite loss, fatigue, nausea, and diarrhea showed greater increases over time in patients with follow-up cachexia. Because we cannot exclude the possibility that the effects of chemotherapy, especially multidrug chemotherapy, overcame any negative effects of cancer cachexia itself, future studies may need to focus on individual chemotherapeutic regimens.

## Electronic supplementary material

ESM 1 (DOCX 37 kb)

## Data Availability

Qualified researchers may request Ono Pharma to disclose individual patient-level data from clinical studies through the following website: https://clinicalstudydatarequest.com/. For more information on Ono Pharma’s Policy for the Disclosure of Clinical Study Data, please see the following website: https://www.ono.co.jp/eng/rd/policy.html
