# Supplementary material for: Incidence and frequency of cancer cachexia during chemotherapy for advanced pancreatic ductal adenocarcinoma
Source: Support Care Cancer. 2020 Feb 26;28(11):5271–9. doi: 10.1007/s00520-020-05346-8 (PMC7546994; doi:10.1007/s00520-020-05346-8)
Supplement: Supplementary file 1 — (DOCX 37 kb) [file 520_2020_5346_MOESM1_ESM.docx]

# Supplementary materials

**Table S1.** Characteristics of patients with or without follow-up cachexia during first-line chemotherapy

|  | Patients with follow-up cachexia | Patients without follow-up cachexia | |
| --- | --- | --- | --- |
| N | 65 | 85 | |
| Sex |  |  | |
| Male | 41 (63.1%) | 47 (55.3%) | |
| Female | 24 (36.9%) | 38 (44.7%) | |
| Age, years | 65.0 (35–83) | 64.0 (37–79) | |
| BMI, kg/m^2^ | 21.16 (13.8–32.5) | 21.92 (14.9–33.3) |  |
| Primary site |  |  | |
| Pancreatic head | 31 (47.7%) | 29 (34.1%) | |
| Pancreatic body | 30 (46.2%) | 33 (38.8%) | |
| Pancreatic tail | 4 (6.2%) | 23 (27.1%) | |
| UICC stage |  |  | |
| III | 23 (35.4%) | 21 (24.7%) | |
| IV | 42 (64.6%) | 64 (75.3%) | |
| Modified Glasgow prognosis score* |  |  | |
| A | 42 (64.6%) | 54 (63.5%) | |
| B | 5 (7.7%) | 2 (2.4%) | |
| C | 13 (20.0%) | 25 (29.4%) | |
| D | 5 (7.7%) | 4 (4.7%) | |
| First-line chemotherapy |  |  | |
| Modified FOLFIRINOX | 25 (38.5%) | 20 (23.5%) | |
| Gemcitabine monotherapy | 16 (24.6%) | 41 (48.2%) | |
| Gemcitabine + nab-paclitaxel | 24 (36.9%) | 24 (28.2%) | |
| Cancer cachexia at the start of first-line chemotherapy (base cachexia), yes | 38 (58.5%) | 37 (43.5%) | |
| Cancer cachexia at the start of first-line chemotherapy (base cachexia), no | 27 (41.5%) | 48 (56.5%) | |
| Comorbidities |  |  | |
| Hypertension | 21 (32.3%) | 25 (29.4%) | |
| Diabetes mellitus | 19 (29.2%) | 19 (22.4%) | |
| Dyslipidemia | 2 (3.1%) | 7 (8.2%) | |
| Other | 8 (12.3%) | 14 (16.5%) | |
| None | 30 (46.2%) | 43 (50.6%) | |
| CrCl^†^, mL/min | 84.214 (42.97–141.80) | 84.306 (36.30–177.05) | |
| Neutrophil/lymphocyte ratio | 2.623 (1.19–8.84) | 3.115 (0.81–12.68) | |
| CA19-9, U/mL | 560.70 (0.6–197100.0) | 751.00 (0.2–284200.0) | |
| Sodium, mmol/L | 141.0 (131–144) | 141.0 (131–144) | |
| Potassium, mmol/L | 4.30 (3.4–5.5) | 4.30 (3.6–6.3) | |
| AST, U/L | 22.0 (11–83) | 22.0 (11–136) | |
| ALT, U/L | 22.0 (9–137) | 24.0 (7–187) | |
| ALP, U/L | 330.0 (104–1997) | 320.0 (106–2558) | |
| Cholinesterase, U/L | 258.0 (109–367) | 288.5 (165–553) | |
| Total bilirubin, mg/dL | 0.700 (0.12–1.70) | 0.600 (0.29–2.20) | |
| Neutrophil count, cells/µL | 3710.0 (1510–9990) | 3830.0 (1620–12650) |  |
| WBC count, cells/µL | 6100.0 (2600–12500) | 5800.0 (2500–16100) |  |
| Lymphocyte count, cells/µL | 1330.0 (690–2770) | 1310.0 (440–2470) |  |
| Platelet count, ×10^4^ cells/µL | 18.60 (11.0–52.4) | 18.50 (8.5–35.9) |  |
| Creatinine, mg/dL | 0.680 (0.33–1.00) | 0.640 (0.40–1.34) |  |
| Albumin, g/dL | 3.90 (2.7–4.6) | 4.00 (2.8–4.9) |  |
| Total protein, g/dL | 6.90 (6.0–8.1) | 6.90 (5.6–8.1) |  |
| CRP, mg/dL | 0.350 (0.02–9.35) | 0.450 (0.01–9.94) |  |
| Hemoglobin, g/dL | 12.60 (8.1–18.2) | 12.60 (8.5–15.6) |  |
| Glucose, mg/dL | 107.0 (64–266) | 109.0 (59–350) | |

Values are number (percent) of patients or median (range)

*A = albumin ≥3.5 g/dL and CRP <1.0 mg/dL; B = albumin <3.5 g/dL and CRP <1.0 mg/dL; C = albumin ≥3.5 g/dL and CRP ≥1.0 mg/dL; D = albumin <3.5 g/dL and CRP ≥1.0 mg/dL

^†^Cockcroft–Gault formula

BMI, body mass index; UICC, Union for International Cancer Control; COPD, chronic obstructive pulmonary disease; CrCl, creatinine clearance; CA19-9, carbohydrate antigen 19-9; AST, aspartate aminotransferase; ALT, alanine aminotransferase; ALP, alkaline phosphatase; WBC, white blood cell count; CRP, C-reactive protein

**Table S2.** The changes of various laboratory variables from baseline to 12, 24, or 48 weeks in patients with and without cancer cachexia occurring within 24 weeks after starting first-line chemotherapy

| Variable | Patients with follow-up cachexia | | | Patients without follow-up cachexia | | | *P** |
| --- | --- | --- | --- | --- | --- | --- | --- |
|  | n | Median (range) | Mean (SD) | n | Median (range) | Mean (SD) |  |
| CRP (mg/dL) | | | | | | | |
| 12 W | 68 | 0.000 (−9.19 to 11.62) | 0.203 (3.067) | 82 | 0.105 (−7.20 to 12.35) | 0.768 (2.938) | 0.2520 |
| 24 W | 63 | 0.110 (−9.23 to 23.29) | 1.725 (4.421) | 69 | 0.080 (−7.37 to 24.37) | 0.665 (3.372) | 0.1220 |
| 48 W | 28 | 0.280 (−9.19 to 8.45) | 0.548 (3.081) | 52 | 0.580 (−3.64 to 20.33) | 3.039 (6.027) | 0.0443 |
| Albumin (g/dL) | | | | | | | |
| 12 W | 68 | −0.30 (−2.0 to 0.9) | −0.30 (0.54) | 82 | −0.30 (−1.9 to 0.8) | −0.36 (0.50) | 0.4692 |
| 24 W | 63 | −0.40 (−2.8 to 0.8) | −0.48 (0.73) | 69 | −0.30 (−1.3 to 0.7) | −0.29 (0.44) | 0.0666 |
| 48 W | 29 | −0.30 (−2.0 to 0.8) | −0.40 (0.69) | 52 | −0.55 (−3.1 to 0.9) | −0.59 (0.78) | 0.2802 |
| Neutrophil count (/μL) | | | | | | | |
| 12 W | 68 | −550.0 (−8580 to 19600) | 38.4 (4305.0) | 82 | −605.0 (−5970 to 18270) | 302.2 (4314.9) | 0.7096 |
| 24 W | 63 | 120.0 (−7200 to 14040) | 515.7 (3348.2) | 69 | −540.0 (−4660 to 33200) | 87.0 (4781.4) | 0.5552 |
| 48 W | 29 | −240.0 (−7440 to 4600) | −30.3 (2239.9) | 52 | 310.0 (−5170 to 9880) | 798.3 (2724.6) | 0.1670 |
| Lymphocyte count (/μL) | | | | | | | |
| 12 W | 68 | −45.0 (−1380 to 2070) | 6.0 (565.0) | 82 | −90.0 (−1140 to 790) | −80.0 (342.9) | 0.2529 |
| 24 W | 63 | −90.0 (−1490 to 1520) | −134.9 (472.6) | 69 | −70.0 (−930 to 1270) | −50.6 (438.4) | 0.2894 |
| 48 W | 29 | −270.0 (−1530 to 640) | −278.6 (520.1) | 52 | −110.0 (−960 to 1410) | −55.4 (500.9) | 0.0615 |

*t-test

SD, standard deviation; CRP, C-reactive peptide; W, weeks
